# Nutritional Management in Bariatric Surgery Patients

**DOI:** 10.3390/ijerph182212049

**Published:** 2021-11-17

**Authors:** Andrea Deledda, Stefano Pintus, Andrea Loviselli, Michele Fosci, Giovanni Fantola, Fernanda Velluzzi

**Affiliations:** 1 Endocrinology and Obesity Unit, Department of Medical Sciences and Public Health, University of Cagliari, 09124 Cagliari, Italy; andredele@tiscali.it (A.D.); alovise2@gmail.com (A.L.); fernandavelluzzi@gmail.com (F.V.); 2 Obesity Surgery Unit, Department of Surgery, ARNAS G. Brotzu, 09134 Cagliari, Italy; stepintuss@gmail.com (S.P.); giovannifantola@aob.it (G.F.)

**Keywords:** obesity, bariatric surgery, metabolic surgery, nutritional deficiencies, nutrition pre-habilitation, nutrition care, dietary supplements, diabetes remission

## Abstract

The obesity epidemic, mainly due to lifestyle changes in recent decades, leads to serious comorbidities that reduce life expectancy. This situation is affecting the health policies of many nations around the world. Traditional measures such as diet, physical activity, and drugs are often not enough to achieve weight loss goals and to maintain the results over time. Bariatric surgery (BS) includes various techniques, which favor rapid and sustained weight loss. BS is a useful and, in most cases, the best treatment in severe and complicated obesity. In addition, it has a greater benefit/risk ratio than non-surgical traditional therapies. BS can allow the obese patient to lose weight quickly compared with traditional lifestyle changes, and with a greater probability of maintaining the results. Moreover, BS promotes improvements in metabolic parameters, even diabetes remission, and in the quality of life. These changes can lead to an increase of life expectancy by over 6 years on average. The nutrition of people before and after BS must be the subject of indications from a trained staff, and patients must be followed in the subsequent years to reduce the risk of malnutrition and the associated problems. In particular, it is still debated whether it is necessary to lose weight prior to surgery, a procedure that can facilitate the surgeon’s work reducing the surgical risk, but at the same time, lengthens preparation times increasing the risks associated with concomitant pathologies. Furthermore, preventing nutritional deficiencies prior to the intervention can improve the results and reduce short- and long-term mortality.

## 1. Introduction

Obesity is considered a chronic and progressive clinical condition, characterized by an excessive or abnormal accumulation of body fat that negatively affects health [1], with an increasing prevalence worldwide [2]. Several factors, such as genetic, epigenetic, microbial, social, and environmental factors concur to its complex etiopathogenesis, resulting in a persistent energy imbalance between the caloric intake and the energy expenditure [3]. In this regard, the global shift in the food system towards high-calorie, often unhealthy, nutritional patterns [4] and the increase in sedentary behavior, appear to be the main drivers of the obesity pandemic [5]. Nevertheless, many other factors contribute to the development of obesity. In particular, gut microbiota plays a key role in regulating host metabolic processes, showing in obese patients a different pattern from normal weight subjects [6,7]. In addition, the influence of diet on the microbiota composition has been established, and restrictive diets have been shown to decrease the microbiota abundance, due to nutrient deficiency rather than weight loss [8]. On the other hand, less restrictive diets, based on the Mediterranean diet principles, have been shown to positively modulate microbiota composition and diversity [9,10,11].

According to the WHO criteria, the body mass index (BMI), ratio of weight to height squared, have been adopted to define the conditions of overweight (BMI 25–29.9 kg/m^2^) or obesity (BMI > 30 kg/m^2^), and the associated health risk [12,13]. Mortality and morbidity increase as BMI increases. Therefore, patients affected by severe obesity have the highest risk of death and obesity related diseases [14]. However, people classified as overweight or even people with a normal weight (BMI 18.5–24.9 kg/m^2^) but reduced lean mass and excess of visceral fat (sarcopenic obesity), still have a health risk, similar to patients affected by obesity [15]. In fact, BMI is unable to discriminate between the body composition, specifically to define the proportion of fat and lean mass, nor to indicate the fat distribution. For these reasons, despite the usefulness of BMI, it has some limitations at the individual level, while remaining a good tool for the general population [12,15].

Obesity leads to large alterations in the adipose tissue, which is no longer considered an inert storehouse of excess calories, but an endocrine tissue that influences metabolism in different ways [16]. Fat excess modulates the immune system and induces a chronic low-grade inflammation, making the patients affected by obesity particularly prone to developing cardiovascular, respiratory, metabolic, hepatobiliary, neurodegenerative, psychiatric, osteoarticular diseases, some cancers, and in general reducing the quality of life [12,17].

In addition to the clinical issues, obesity represents a major challenge for public health, in the economic aspect. The increasing costs for the management of its complications, including direct (drugs, hospitalizations, related diseases) and indirect costs (loss of days of work, loss of years in health), pose a serious threat to the economy of national health systems [18,19].

In this regard, prevention is fundamental, with a focus on the availability of healthy food options, including their cost reduction through agricultural subsidies, taxation of unhealthy food, and urban planning, which also offers areas dedicated to different types of physical activity [12].

Unfortunately, obesity prevalence is dramatically rising also in the youth, increasing the years of metabolic stress to which the body is subjected, and enhancing the early cardiovascular risk. Childhood obesity frequently persists until adulthood, further reducing the chance of losing weight as adults [20].

The metabolic and cardiovascular aspects of obesity are part of the same problem. The low-grade inflammatory state associated with obesity is a major driver of insulin resistance, the main characteristic of type 2 diabetes pathophysiology, but also contributes to other pathological conditions. Obesity has been associated with a higher prevalence of several forms of cancer including colorectal, pancreatic, kidney, endometrial, postmenopausal breast, and adenocarcinoma of the oesophagus. The impact of obesity in developing cancers is linked to the interaction among obesity-related insulin resistance, excessive nutrients, hyperinsulinemia, sustained hyperglycaemia, oxidative stress, inflammation, and production of adipokines. The wide range of morbidities associated with obesity represents a significant clinical issue for the affected subjects, but these risk factors can be positively modified by weight loss [21].

Estimating energy expenditure in obese patients is a problem which is not easy to solve [22]. In thermodynamic terms, obesity is the result of a constant introduction of more calories than those spent, but at the biological level these calories can undergo a “partitioning”, an address that promotes the accumulation rather than oxidation [23]. For this reason, a previous weight loss, may predispose to a consequent adipose accumulation, to an even greater extent than previously present [24,25]. In this regard, obesity could be considered a cyclic disease with low possibilities of definitive healing. The existence of a weight set-point, regulated at the neuro-humoral level, facilitates the weight regain after a period of caloric restriction, and the lifestyle changes are difficult to maintain in the long term. This makes most of the dietary attempts unsuccessful, and in predisposed individuals, it also leads to a higher final weight than the initial one (weight cycling) [24,25].

Furthermore, overweight and obesity are frequently associated with psychopathological conditions, in a possible bidirectional relationship [26]. This finding has important implications, especially in the obesity management, justifying the need of a careful evaluation [27,28,29].

Finally, another important issue is the stigmatization, commonly observed in different social settings and, unexpectedly, also in health care settings. The patient affected by obesity is frowned upon, considered as weak, lacking will-power, and often a liar person, and this contributes to the reduction of both self-esteem and compliance with treatments [30,31].

Both in controlled trials and in real life, the treatment strategies based on dietary and lifestyle improvements, such as the increase of physical activity level, have shown limited effectiveness, particularly in the medium-long term. This is partly due to the obesogenic environment that surrounds people, as well as to a lack of will and neglect of good habits. However, the neuroendocrine adaptations that favor the recovery of weight loss, inducing more hunger and reducing energy expenditure, seem to be even more important. [12,32].

A few guidelines suggest that it is not possible to identify a diet which is suitable for everyone, especially with regards to the ratio of macronutrients. Moreover, it should be tailored to the health condition and preferences of each patient in order to facilitate the following of the treatment plan and reduce the drop-out [12,32].

However, the use of healthy lower-calorie substitutes in place of the foods normally consumed is emphasized in order to promote a negative energy balance [12].

There have been a large amount of failures in the history of the obesity drug treatment. In general, reduced efficacy, safety problems, reluctance from patients and doctors, and lack of compensation by insurance companies, have reduced their spread. In addition, only in recent years, treatments that appear effective and safe have been approved, but unfortunately are also expensive [12,33].

Bariatric surgery (BS) is currently considered the most effective therapy for long-term weight loss and for the reduction of comorbidities and mortality due to severe obesity [34]. In addition, BS is recommended by obesity guidelines according to the BMI levels and related diseases [35]. Obesity does not necessarily mean a good nutrition state. Nutritional management in the pre-operatory period is still a matter of debate, particularly regarding the nutritional deficiencies frequently observed in obesity and weight loss prior to surgery.

The aim of this narrative review is to analyze the most recent studies on this topic.

## 2. Bariatric Surgery

Bariatric surgery, also called metabolic surgery, induces improvements at the metabolic and hormonal level. It is the treatment of choice for higher-risk patients with obesity, and is considered effective and safe, although the decision should be made based on a risk-benefit analysis. A team of experts is needed for the nutritional, medical, metabolic, and psychological management of patients before and after the surgery [36].

BS is indicated for patients with a BMI > 40 kg/m^2^ or a BMI > 35 kg/m^2^ with comorbidities [12]. In recent years, it has also been applied to patients with lower BMI, especially in the case of uncontrolled diabetes [37]. However, some researchers do not consider BS useful in the case of a BMI between 30 and 35 kg/m^2^, even in the presence of diabetes. At present, there are no indications for BS in overweight individuals [38]. In addition, a retrospective study indicated the usefulness of BS with few adverse events for people with type 1 diabetes and obesity [39]. Finally, a recent American Heart Association (AHA) (Dallas, TX, USA) statement has defined BS as suitable for cardiovascular risk management and atrial fibrillation, since it provides greater benefits than lifestyle changes [40].

BS appears safe even in adolescents between 13 and 17 years. However, in this age group, it is still underused [41]. A 10-year follow-up showed high efficacy on the weight loss and resolution of comorbidities (hypertension, type 2 diabetes, and dyslipidaemia) in most of the children and adolescents that underwent sleeve gastrectomy (SG), without alteration of growth or other safety concerns [42].

In general, the perioperative risk appears similar to other more practiced surgical procedures, such a hysterectomy, appendectomy, and cholecystectomy [43].

In 2013, regarding surgical techniques, the International Federation for the Surgery of Obesity and Metabolic Disorder (IFSO) (Napoli, Italy) reported a worldwide survey that recognized four types of most the common procedures [44]:

Roux-en-Y gastric bypass (RYGB) represents 40% of all surgical procedures. Here, the gastric pouch is fashioned with a volume of 25 cm, gastro-jejunal anastomosis is performed with 100–150 cm of alimentary limb, and jejuno-jejunal anastomosis with 75 cm of a bilio-pancreatic limb. It is considered a metabolic procedure (restrictive and malabsorptive) [45].

Laparoscopic sleeve gastrectomy (LSG) represents 46% of all bariatric procedures worldwide. It consists of a gastric resection fashioned as a sleeve tube. Due to the gastric fundectomy, it is considered a restrictive procedure with metabolic effects [46].

One-anastomosis gastric bypass (OAGB) is an emergent procedure that currently represents more than 7% of all surgical procedures. It consists of a gastric pouch fashioned as a sleeve tube with gastro-jejunal anastomosis. This procedure has great metabolic effects due to a malabsorptive function. The bilio-pancreatic limb is normally 180–220 cm, according to the patient’s BMI [47].

Biliopancreatic diversion (BPD) consists of a horizontal gastric resection combined with the closure of a duodenal stump, gastroileal anastomosis, and an ileoileal anastomosis. At present, this technique is performed less often than the other types of interventions [48].

Although the underlying mechanisms are not completely clarified, the beneficial effects of BS on obesity and the associated diseases are related to the complex interactions between the endocrine, immune, digestive, and nervous systems. Thanks to the changes in these systems, the basic inflammatory state typical of obesity is reduced, while the variation of the gut microbiota and bile acids contributes to metabolic improvements. The improvement in glucose metabolism is mainly linked to a reduced glucose absorption and precocious increase of glucagon-like peptide 1 (GLP-1) levels, due to the faster transit of bolus through the gastric pouch. In addition, an increase of insulin sensitivity, due to the reduction of lipolysis from visceral fat, and the resulting lower release of free fatty acids (FFA) through the portal system into the liver, has been hypothesized [34,49].

BS reduces 5 years of mortality [50], and, particularly RYGB decreases cardiovascular and renal risk [51], more significantly than the non-surgical treatment. However, above all, BS has been shown to be able in most cases to induce remission of type 2 diabetes, a chronic disease that increases mortality in obese patients, especially due to micro- and macrovascular complications. A study in Caucasian populations estimated the remission of diabetes at 63.7% of cases, independently from the starting BMI. A shorter duration of the disease, a lower fasting glycemia, and the absence of insulin therapy, increased the chance of remission after BS [52], while the gastric with diversion procedures (RYGB and BPD) appeared the most effective in inducing the remission [53].

Moreover, BS reduces the general risk of cancer, particularly breast [54,55] and liver cancer [56], and especially in patients with hepatic steatosis [57]. Furthermore, hepatic steatosis improves at least partly, due to the changes in gut microbiota [58]. Nevertheless, in rare cases, the RYGB intervention has shown to favor steatosis and malnutrition [59].

Regarding the gut microbiota, an improvement in the ratio of *Firmicutes* to *Bacteroidetes* and an increase in *A. muciniphila*, commonly altered in obesity, also due to the low dietary intake of fibers and antioxidants [60], are observed. In addition, these changes are related to better health [61]. Despite the fact that these changes are extremely individual among patients, and also depend on the surgery technique, the microbiota composition and functions are modified with developing a greater species diversity [62].

Following BS, the digestion, absorption, and metabolic fate of nutrients and energy expenditure through the brown adipose tissue (BAT) are positively influenced. Other changes, regarding the increased microbial diversity and the energy consumption, with a reduced ability to extract calories, are all features of a leaner phenotype. RYGB appears to be the most increasing diversity intervention [63]. The *phylum* that mostly increased is *Proteobacteria*, while changes in oxygen availability seem to reduce the abundance of *Bifidobacteria* and *Blautia* [64].

Overall, microbiota modification is possibly a notable part of the slimming effect of BS. Although some specific mechanisms are still partially not understood, the beneficial modulating effects on the microbiota profile seem to persist in the long term [65].

On the other hand, in the case of reduced microbiota diversity subsequent to weight loss, the risk of weight-regain increases [63].

The administration of probiotics and prebiotics can be beneficial in supporting diversity and promoting the absorption of micronutrients, as well as improving metabolic and inflammatory markers [64].

Perioperative mortality is closely linked to the functional state of patients, and also to the nutritional status, the topic of this review. According to a retrospective study carried out on more than 44,000 patients, mortality at 30 days was 0.14%. However, in a totally dependent person, it has been estimated to increase by 27 times. In addition, unintentional slimming in the previous 6 months increased the risk by 13 times. Other conditions (male sex, BMI > 50 kg/m^2^, age > 45, diabetes, dyspnoea) have given only a small increase of mortality [66].

On the other hand, a recent meta-analysis estimated the reduction in mortality in obese patients undergoing BS, compared with those who received an usual medical treatment, by 49.2% and the increase in life expectancy by 6.1 years. The benefits increased in diabetic patients [67].

The contraindications of BS are various, but often not absolute and must be evaluated on a case-by-case basis. Uncorrectable coagulopathies or contraindications to anaesthesia, and the presence of severe organ failure or tumours with short life expectancy, do not recommend the procedure. Hyperphagia linked to Prader-Willi syndrome or mental problems give a high risk of failure. In general, mental health must be evaluated and the patient must be informed and motivated for the difficult follow-up. Moreover, an active peptic ulcer is a contraindication [68]. According to a very recent review on obesity and kidney disease, people undergoing BS may have greater complications in the case of severe renal failure [69].

Furthermore, BS has been shown to lower the risk of intrapartum and birth complications [70]. However, it is advisable to delay pregnancy at least 1 year after surgery [71].

Evidently, BS is not risk-free. Both mid- and long-term complications have been described, although determining their incidence is difficult due to the high rate of patients lost to follow-up [72]. Typical problems are intestinal obstruction, marginal ulcer, ventral hernia, and gallstones. Examples of metabolic-related complications are nephrolithiasis, osteoporosis, and hypoglycaemia. Mineral and vitamin deficiencies often occur. Micronutrient deficiencies following gastric bypass include: Iron, 33% to 55%; calcium/vitamin D, 24% to 60%; vitamin B_12_, 24% to 70%; copper, 10% to 15%; and thiamine, <5% [73]. To prevent or reduce the incidence of these complications, the routine nutrient supplementation including multivitamins, vitamin B_12_, iron, minerals, calcium, and vitamin D is recommended [74].

Nutritional B vitamins deficiency post BS exposes patients to a high risk of developing mental, cognitive, and neurological complications. Therefore, a persistent supplementation is required. In addition, hyperhomocysteinemia can be a driver of these conditions [75]. A small amount of BS patients experience other types of mental impairment and addictions (i.e., alcohol, opioids) that can be linked to hormonal and bile acids imbalance [76].

Bone fractures [77] and anaemia [78] are also risks linked to BS. Fractures are favored by mixed and malabsorptive procedures, while RYGB is more linked to anaemia. Both the aforementioned studies underline the relevance of compliance for supplements after the procedures.

Patients must be kept under control in subsequent years to avoid risks, in particular related to malnutrition [79]. Moreover, significant caution is necessary to avoid common intestinal symptoms, such as flatulence and diarrhoea, early and late dumping syndrome, constipation, dysphagia, vomiting, food intolerances, and dehydration.

Table 1 shows a glossary of medical terms reported in the present section.

Regarding the outcomes, a recent meta-analysis of adult patients estimated a fat mass (FM) loss at 12 months of 28.9 kg with RYGB, 20.81 kg with LSG, and 18.51 with gastric banding (GB) [80].

Fat loss is more sustained and persistent in the first year after surgery, with no evidence of plateau [81]. Furthermore, BS can induce a better adipose tissue morphology, enhancing hyperplasia and decreasing hypertrophy of adipocytes, which in turn could contribute to the improvement of insulin sensitivity [49].

Another meta-analysis shows the results achieved in adolescents: The short-term weight loss (after 6 months) was estimated at an average of −5.4 BMI points after gastric band, −11.5 after gastric sleeve, and −18.8 after gastric bypass, while at mid-term (36 months), it was −10.3, −13.0, and −15.0, respectively [82].

Although the trend is lower than observed with the conventional approaches, such as lifestyle or pharmacological interventions, even BS patients may tend to recover weight. According to the results reported by Velapati et al. [83], 93% of patients maintained at least 10% of the weight loss from baseline, 70% maintained at least 20% of the weight loss, and only 40% maintained at least 30% of the weight loss after 12 years. These results are still better than those obtained with the medical intensive treatment alone [84].

Finally, BS appears to be cost-effective, compared to the usual non-surgical therapy for obesity and the related chronic diseases, in terms of drugs, hospitalization, and work days lost, at least in high-income countries [85].

## 3. Presurgical Nutritional Deficiencies

One of the most important aspects in the medical management of the bariatric patient refers to nutritional management. Despite their appearance, obese patients often present nutritional deficiencies both in terms of micronutrients [86,87,88,89] and body composition [90].

The assessment of the nutritional status of patients, prior to undergoing BS, plays an important role in the post-surgical management. During the last few years, several studies reported that patients with severe obesity often display micronutrient deficiencies (MDs) when compared with normal weight controls. It is estimated that 20–30% of the candidates for BS have MDs prior to surgery [91].

Comparing a group of severely obese patients with normal weight subjects, Asheim showed significantly lower concentrations of vitamins A, B_6_, C, 25-hydroxyvitamin D (25(OH)D), and vitamin E [86]. In 2014, another study including 200 patients with severe obesity found low levels of iron in 38% of the patients, low serum folate in 24%, and low serum B_12_ in 11%, while 81% had hypovitaminosis D [87]. Finally, in 2017, Krzizek demonstrated that 63.2% of patients had a deficit in folic acid (<5.3 ng/mL), 97.5% in (25(OH)D) (<75 nmol/L), and 30.2% had a parathyroid hormone (PTH) elevation (>56.9 pg/mL). A total of 5.1% patients presented with a deficit in vitamin B_12_ (<188 pg/mL), 6.2% in vitamin A (<1.05 μmol/L), and 9.6% exhibited iron deficiency (ferritin < 15 μg/L) [92]. Cobalamin deficiency can be related with several drugs largely used for obesity comorbidities, such as metformin, proton pump inhibitors, angiotensin converting enzyme inhibitors, colchicine, other than small bowel bacterial overgrowth [93]. The iron deficiency among obese people (as high of 45% in some studies) [94] can be ascribed to several causes: Poor dietary intake, increased iron requirement, and impaired absorption in the duodenum [95,96]. The proinflammatory adipokine hepcidin rises in obesity due to the chronic low-grade inflammation present in this pathology. The result is a decreased expression of ferroportin, an iron transporter that favors its absorption in the small bowel. Furthermore, high levels of hepcidin inhibit the release of recycled iron from macrophages and prevent the mobilization of stored iron from hepatocytes [97]. In adults, a consistent association has been found between low magnesium status, often seen in obese patients [98], insulin resistance, and metabolic syndrome [99,100]. There is inadequate data on the thiamine status in the general obese population. However, in patients undergoing BS, the prevalence of thiamine deficiency is estimated to be between 15.5 and 29% in the preoperative group with a significantly higher prevalence among Hispanics and African-Americans as compared to Caucasians [101,102]. The prevalence of preoperative vitamin A deficiency in BS candidates is 2–17%. A preoperative deficiency may be particularly problematic with a malabsorptive procedure, such as RYGB, as the risk for poor absorption of vitamin A increases further after surgery [88,103,104].

The potential consequences of vitamin D deficiency in patients undergoing BS, considering the resulting increased risk for postoperative deficiency, are significant. Although BS involves generally safe procedures, major complications following the surgery include poor wound healing and heightened risk for infection. Vitamin D, as discussed, plays a major role in each of these mechanisms, which are already more frequent in obesity [105,106,107,108].

Obese patients have been found to have lower zinc concentrations in the plasma and erythrocytes, as well as higher urinary excretion as compared to lean subjects [109,110]. Zinc deficiency can be seen in up to 50% of patients undergoing BS prior to the procedure. Zinc plays a significant role in the biosynthesis and action of insulin and its deficiency has been linked to metabolic syndrome.

Therefore, the assessment and correction of the nutritional status prio to the procedure in BS candidates is essential for the prevention of post-bariatric MDs. Moreover, preventing post bariatric MDs is considered a major issue since they can favor weight regain, which stimulates food craving [79].

In 2019, Schiavo et al. [111] reported that patients who received a preoperative MDs correction did not develop new MDs in the first year after BS, whereas all of the patients who did not receive MDs correction prior to BS, continued to be deficient in one or more micronutrient after surgery, despite postoperative supplementation.

In patients with obesity, MDs could be attributed to poor-quality, high-calories, and high-fat diet [112]. In addition, they can interfere with energy metabolism [113]. For example, excessive amounts of simple sugar, and in general junk food could lead to a deficit of vitamin B_1_ [89]. Moreover, the iron status could be affected by adipose tissue inflammation and increased expression of the systemic iron regulatory proteins [90]. Furthermore, the adipose tissue could play a role in the storage of lipophilic molecules as vitamin D, which explains the difference between the levels in normal-weight people compared with obese patients [114].

Obesity may itself result in malabsorption of certain nutrients [115]. Another cause may be related to the frequent attempts to lose weight by adopting various crash diet programs with no regard for nutritional adequacy.

## 4. Weight Loss Strategies Prior to Bariatric Surgery

The purpose of preoperative weight loss prior to BS is mainly to improve the operative and postoperative outcomes (morbidity and mortality) in the mid- and long-term (more than 1 year after BS) [116]. However, the actual need is still a matter of debate.

In this regard, various national guidelines exist. For example, the Canadians guidelines recommend a 10% weight loss in the 6 months prior to surgery [117]. In addition, in the United States, many insurance companies require a preoperative weight loss of 5–10% [118]. The expected benefits of preoperative weight loss include the selection of patients that are most motivated to undergo the surgical intervention, the education in postoperative dietary restrictions, the reduction in perioperative morbidity, and the reduction of liver size and surgical time [119].

On the other hand, the most relevant international guidelines do not provide any clear indication on preoperative weight loss. Indeed, those guidelines agree that a period of identifiable medical management is necessary in all of the patients prior to BS and that it is also necessary to assess the motivation and willingness of patients to adhere to follow-up programs. However, the preoperative weight loss is never mentioned, neither in the indication for BS, nor in the preoperative evaluation [34,74,120].

The National Institute of Health statement does not consider weight loss as mandatory [121]. Despite the fact that weight loss, which makes the surgical procedure easier, is universally accepted by surgeons, there is no evidence in the literature that this can improve the outcomes.

Cassie et al. [116] analyzed 27 studies in a systematic review that also included two randomized prospective studies [122,123]. The first endpoint of the review was weight loss. Nine studies reported a positive effect of the preoperative weight loss on the weight drop to 6, 12, 24 or 36 months. In particular, the two randomized trials reported an excess weight loss percentage (EWL%) over 6 months, of 53.9% in the preoperative weight loss group and of 50.9% in the control group [122], as well as an EWL% over 12 months of 78.4% versus 71.3% [123]. However, these differences were not significant. Moreover, Alami et al. [122] reported an improvement in the operating times (average −37.4 min) in patients with preoperative weight loss. The analysis included 11 studies, but only two (involving 1234 patients) showed a significant reduction in complications with preoperative weight loss. The review concludes that the evidence to support a preoperative weight loss is currently scarce [116].

A retrospective analysis on 480,075 patients showed that preoperative weight loss reduces 30-days mortality after BS [124]. In addition, it leads to the improvement of a few significant outcomes [125]. However, a mandatory slimming regimen could result in the loss of candidates, and potentially expose these patients to greater risks.

On this issue, a recent meta-analysis failed to show the clear benefits. Therefore, the choice should be made by the medical team [126].

Several studies with positive results in terms of weight loss have been performed, treating patients with the very low-calorie diet (VLCD) or intragastric balloon placement [127,128].

The latest guidelines by the European Association for the Study of Obesity (EASO) (Teddington, UK) on ketogenic diet for obesity, suggest that the very low-calorie ketogenic diet (VLCKD) is a suitable approach prior to BS [129], while a classic balanced low-calorie diet (LCD) does not appear as useful, according to the review by Bettini et al. [130].

Albanese et al. [131] reported that in the 178 patients submitted to SG, VLCKD showed better results than VLCD on the surgical outcome, influencing the drainage output, postoperative haemoglobin levels, and hospital stay. However, no data on the reduction of liver and visceral fat volumes were obtained in this study.

In another similar study, VLCKD ensured a greater weight loss compared with VLCD, without differences in liver volumes, clinical biochemical parameters, rate of surgical complications, and hospital length stay, after 21 days of diet [132].

In another study, a classic VLCD showed the loss of an excess of lean mass [133] and this is known as a negative prognostic factor on the healthy state, quality of life, and weight loss maintenance [134,135]. Although in the context of BS, VLCD is not considered a major factor for weight regain, it is possibly an underappreciated one [136].

Furthermore, data from a systematic review assert that liver size reduction and weight loss are not necessarily linked to a higher caloric restriction, and the results on operative risk reduction are unclear [137].

Finally, although there is no consensus on which diet is the best prior to BS, it should not last for more than 3 months in order to not lose motivation and compliance [79].

Different considerations could be made in the case of patients with super obesity (BMI > 50 kg/m^2^), which are considered by the surgeon as complex patients. Regardless of the type of surgery, super obesity exposes patients to amplified perioperative risks. The hepatomegaly, particularly regarding the left liver lobe, the enhanced visceral fat, and the increased abdominal wall thickness, make the operating zone difficult to access, putting the feasibility and safety of the intervention at risk [138]. The surgical risk, which is primarily haemorrhagic, is accompanied by the risk of respiratory anaesthesiology with difficulty in intubation and ventilation. This is often improved by the reduction of certain parameters, such as the increased neck circumference [138,139].

Other literature data indicate that in the case of super obesity, the result of surgery is less predictable and more frequently prone to failure (lack of weight loss or weight regain) [140]. In fact, these patients require more aggressive interventions in terms of malabsorption. Moreover, these interventions are characterized by a greater technical difficulty.

The literature shows that all of the restrictive interventions (RYGB and SG), which are technically more feasible in superobese patients, do not give the same results as the interventions with a malabsorptive nature. Bettencourt-Silva et al. [140] in a recent retrospective observational study, analyzed 213 superobese patients undergoing BS with three different techniques: GB, RYGB, and SG. After 1 and 2 years of follow-up, the patients undergoing malabsorptive component (RYGB) surgery had a significantly greater weight loss (BMI; waist to hip ratio; EWL%; TWL%), compared with those subjected to predominantly restrictive component interventions (GB and SG). However, the mean BMI values in patients undergoing RYGB were still above 30 kg/m^2^ (34.52 kg/m^2^ ± 4.58 after 1 year and 33.22 kg/m^2^ ± 4.66 after 2 years). The RYGB intervention is considered more complex and at greater risk, even if the data show no differences in postoperative morbidity. In addition, the follow-up of this study is too short for a long-term evaluation of the three procedures. In fact, super obesity, even more than the less severe obesity, should be considered a chronic pathology with a high risk of weight recovery after 2 years from the intervention.

These considerations have led BS to include several stages in the clinical path of the superobese patient. The first stage would be focused on the preoperatory weight loss in order to reduce surgical risk. The second stage would be aimed at strengthening weight loss, improving the related pathologies, and ensuring the maintenance of long-term weight loss (5 years). In addition, new hospitalization methodologies, such as enhanced recovery after surgery (ERAS), which are designed to ensure a better perioperative recovery, require weight loss to optimize the patient’s clinical picture. This condition is even more valid in a superobese patient [141].

In a recent meta-analysis, Lee et al. [142] examined 13 studies which include three main preoperative clinical pathways under conditions of super obesity. These pathways are called “bridging” since they point to the achievement of an adequate weight loss in order to deal with the ultimate surgery. Six studies with 209 enlisted patients indicated SG as a bridge, two studies with 35 patients indicated the liquid low-calorie diet, and two studies with 35 patients indicated the intragastric balloon. The primary outcome was the difference of BMI before and after the bridge: SG (*p* < 0.0001) and liquid low-calorie diet (*p* = 0.0006), but not the intragastric balloon, which demonstrated a significant BMI reduction. In this case, it should be considered that SG is the only invasive bridge surgery procedure, thus exposing patients to the risk of complications and, in view of their fragility due to super obesity, to enhanced mortality risk. Moreover, the results indicate that the low-calorie liquid diet showed excellent results, although lower than those obtained with SG, which are satisfactory and far superior to the intragastric balloon. In these patients, the preoperative diet allowed the reduction of weight, in order for the single time intervention to be undertaken with less risk. Furthermore, it should be considered that the diet is a preoperative path, which is more prone to failure, especially in the view of the patient’s compliance [142].

## 5. Limitations

The main limitation of this review is the narrative and not systematic model, due to the difficulty in finding homogeneous data on this topic, particularly on the need of weight loss prior to surgery. Most of the studies considered were retrospective, not randomized, and heavily influenced by health insurance directives. Different panels of clinical tests required by different teams prior to surgery have been another limitation for a systematic evaluation.

## 6. Conclusions

Bariatric surgery appears to be the best method for the reduction of disease risk and mortality in patients with severe obesity, as well as in those with concomitant metabolic diseases, such as diabetes. BS has been shown to have a better risk/benefit ratio both in health and economic terms than lifestyle and drug-based interventions, with greater efficacy in the long term.

People who are candidates for BS often have an unbalanced diet, rich in refined foods, added sugars, and fats, that promote nutritional deficiencies and poor health. In particular, hypovitaminosis D, hypocobalaminemia, and the deficiency of some minerals such as zinc and magnesium, often favored also by some drugs, increase the risk of surgical and/or nutritional deficiencies in the long term. Therefore, these deficiencies must be identified and corrected, possibly with supplements, prior to surgery. The need to lose weight prior to surgery in order to reduce complications is still a matter of discussion. Here, the main guidelines do not consider it mandatory to impose weight loss in the preoperative period. This is due to the fact that it could favor the loss of some candidates for surgery and increases the risk of developing comorbidities. However, surgeons agree that a reduction in visceral fat and liver size can ease the operation and reduce surgical risks, especially in superobese patients.

Moreover, there is no agreement on the type of diet to follow. However, in general, a VLCD or a VLCKD diet that promotes rapid weight loss, can be recommended. Furthermore, the development of guidelines that provide specific indications for personalized presurgical management would be desirable.

## Figures and Tables

**Table 1:** Glossary of symptoms and medical conditions related to the “Bariatric Surgery” section.

Medical Term	Definition
Anaemia	deficiency of red cells or of haemoglobin in the blood
Appendectomy	surgical operation to remove the appendix
Atrial fibrillation	heart disease characterized by an irregular and fast heart rate
Cholecystectomy	surgical operation to remove the gall bladder
Diabetes Mellitus	chronic disease characterized by elevated levels of blood sugar
Dumping syndrome	disorder characterized by food that moves too quickly from the stomach into the small bowel
Dyslipidaemia	disease characterized by too high or low blood lipid levels
Dysphagia	difficulty or discomfort in swallowing
Dyspnoea	difficult or labored breathing
Gallstones	small stones that form in the gallbladder
Hepatic steatosis	condition in which excess fat builds up in the liver
Hernia	condition in which an organ pushes through an opening in the muscle or tissue that holds it in place
Hyperhomocysteinemia	condition characterized by high level of homocysteine in the blood
Hyperphagia	abnormally increased appetite for consumption of food
Hypertension	condition characterized by abnormally high blood pressure
Hypoglycaemia	condition in which the blood sugar level is lower than normal
Hysterectomy	surgical operation to remove all or part of the uterus
Nephrolithiasis	condition characterized by stones within the kidney
Osteoporosis	disorder characterized by bone fragility and consequent increase in fracture risk
Peptic ulcer	sore on the lining of stomach or duodenum
Prader-Willi syndrome	genetic disease characterized by physical, mental, and behavioral problems

## Data Availability

Not applicable.

## Abbreviations

25(OH)D	25-hydroxyvitamin D
BAT	Brown Adipose Tissue
BMI	Body Mass Index
BPD	Biliopancreatic diversion
BS	Bariatric Surgery
ERAS	Enhanced Recovery after Surgery
EWL%	Excess Weight Loss Percentage
GB	Gastric Banding
LSG	Laparoscopic Sleeve Gastrectomy
LCD	Low Calorie Diet
MDs	Micronutrient Deficiencies
OAGB	One-anastomosis gastric bypass
RYGB	Roux-en-Y Gastric Bypass
SG	Sleeve Gastrectomy
TWL%	Total Weight Loss Percentage
VLCD	Very Low Calorie Diet
VLCKD	Very Low Calorie Ketogenic Diet
